# Emotion Regulation and Expression Among Parents of Children Residing in Emergency Housing

**DOI:** 10.1007/s10826-026-03360-8

**Published:** 2026-08-24

**Authors:** Daniel Valerio Montero, Alyssa R. Palmer, Ann S. Masten, Madelyn H. Labella

**Affiliations:** 1 Department of Psychological Sciences, William & Mary, 540 Landrum Drive, Williamsburg, VA USA; 2 Department of Psychology, University of Utah, 380 S. 1530 E., Utah 84112 Salt Lake City, United States; 3 Institute of Child Development, University of Minnesota, 51 East River Road, Minneapolis, MN USA

**Keywords:** Emotion regulation, Emotion expression, Parental emotion socialization, Sociodemographic risk, Homelessness

## Abstract

Effective emotion socialization – including parental modeling of healthy emotional expression and adaptive regulation – may support resilience for children experiencing adversity. Unfortunately, parental modeling of emotion may be negatively altered in the context of stress. The current study examined associations among parents’ emotional experience, expression, and regulation in families with young children experiencing homelessness. Participants were 108 primary caregivers (89.8% female) of 4- to 6-year-old children (58.2% male) in emergency housing. Parents reported on their own recent emotional experiences, habitual emotion expressiveness in the family environment, and emotion regulation strategy use (e.g., rumination, positive reappraisal). Parents’ emotional expressions were also coded observationally from video-recorded parent-child interaction tasks. We hypothesized that parental emotional experience and regulation would each predict emotion expressions in the parent-child context, and that parental emotion regulation would moderate the links between the experience and expression of negative emotion. Hypotheses were partially supported. Self-reported subjective emotional experiences predicted self-reported but not observed emotion expressiveness, and associations linking regulation with emotional experience and expressiveness were largely valence-specific (i.e., adaptive regulation predicting positive emotions, maladaptive regulation predicting negative emotions). Adaptive (but not maladaptive) regulation uniquely predicted more positive emotion expressiveness (observed and self-reported) and less self-reported negative expressiveness. Sociodemographic risk was not related to parental emotion variables, and regulation did not moderate links between emotional experience and expression. Results suggest that adaptive regulatory strategies may help parents experience and express more positive and less negative emotions in the context of poverty and homelessness, with potential downstream benefits for children’s social-emotional development.

## Highlights

This study assesses links among self-reported and observed emotion processes among parents of young children in emergency housing.

Self-reported emotion experience predicted self-reported (but not observed) expressiveness in valence-specific ways.

Self-reported emotion regulation strategy use was associated with multiple measures of emotion experience and expressiveness.

Adaptive regulatory strategies predicted more positive and less negative expressiveness, controlling for emotion experience.

Regulatory strategy use did not moderate links between emotion experience and expression.

Children who experience extreme poverty are at risk for a range of adversities that may negatively impact their development (Evans et al., 2013). These risks are likely compounded for families experiencing homelessness, among whom chronic stresses related to economic adversity are enhanced by acute housing insecurity (Wiewel & Hernandez, 2021). Consistent with occupying the extreme end of a continuum of cumulative risk, families experiencing homelessness are at greater risk of negative mental health outcomes compared to those who experience poverty but are stably housed (Bassuk, 2010). Importantly, high-quality parenting may serve as a protective factor against poverty-related risk (Kim-Cohen et al., 2004; Masten & Labella, 2016; Masten & Palmer, 2019; Miller et al., 2011). Multiple aspects of high-quality parenting (e.g., sensitivity, warmth, and effective coregulation) have been linked to better outcomes among toddlers and preschoolers residing in emergency housing; moreover high-quality parenting also has demonstrated moderating effects, buffering children against negative sequelae associated with risk and adversity (Herbers et al., 2011; 2014; Labella et al., 2019; Narayan et al., 2015; Palmer et al., 2020).

Parental emotion socialization – the process by which parents communicate cultural norms for the expression and regulation of emotion (Eisenberg, 2020) – is one aspect of parenting that may contribute to social-emotional adjustment in families experiencing homelessness. Beyond explicit instruction and direct responses to children’s emotions, emotion socialization occurs implicitly through parental modeling of emotion expression and regulation. Early childhood is a particularly critical stage for the socialization of emotional competence, given rapid maturation in regulatory capacities in the first five years of life (e.g., Sala et al., 2014) and heightened demand on children’s social-emotional skills during the transition to formal schooling. Given that young children typically spend more time in proximity to parents compared to school-age children and adolescents, they may be particularly susceptible to observational learning of emotion norms through parental modeling (Morris et al., 2017; Schoppmann et al., 2018).

However, parental emotion modeling may be altered under conditions of acute stress, during which strong negative emotions are elicited more frequently and regulatory capacities may be taxed. E motion expression and experience are distinct constructs (Gross, 2015), and regulatory skills are required to downregulate the corresponding behavioral response of a strong emotional reaction to facilitate social interactions or goal-directed behavior. Parents who maintain healthy emotion expression and regulation during an episode of homelessness may be able to buffer their children from the effects of acute and chronic adversity. For example, one study of families experiencing homelessness found parental warmth and negativity when discussing their children predicted children’s observed positive and negative affect during parent-child interactions as well as their prosocial behavior at school (Labella et al., 2016). Overall, theory and research suggest that the emotional aspects of parenting may be particularly relevant for children’s adaptive functioning in the context of homelessness.

Unfortunately, empirical research rarely includes unhoused populations in studies of emotion socialization, providing an incomplete picture of how the emotion socialization framework applies to racial and ethnic minorities in impoverished and housing-insecure populations (U.S. Department of Housing and Urban Development, 2024). Research examining emotion socialization processes is needed to inform preventive interventions that support resilience among families experiencing housing instability. The present study aimed to address this gap in literature by testing associations among emotional experiences, expression, and regulation among parents experiencing homelessness.

## Parental Emotion Expression in the Context of Poverty-Related Risk

Parental emotion expression is the outward demonstration of internal emotional experiences and is often measured through two methods: (1) parents’ self-reported patterns of habitual expression of emotions in the context of family interactions (e.g., Carona et al., 2021; Halberstadt et al., 1995) and (2) observer ratings of parental affect during research paradigms (e.g., Heller & Baker, 2000; Kim-Cohen et al., 2004; von Suchodoletz et al., 2011). Research suggests that children’s emotional development is optimized when parents model moderate to high levels of positive emotion and relatively low levels of negative emotions. Parental warmth and positive affect have been linked to better social-emotional adjustment in studies involving self-reports (e.g., Clark & Frick 2016; Kim-Cohen et al., 2004; von Suchodoletz et al., 2011), observations (e.g., Eisenberg et al., 2001; Lunkenheimer et al., 2011), and multi-method assessments (e.g., Alley et al., 2025; Davidov & Grusec, 2006; Yavuz et al., 2022) of parental emotion expression and/or warmth. Additionally, self-reported parent negative emotions have been linked to peer problems and externalizing behaviors (e.g., Heller & Baker, 2000).

Unfortunately, as indicated above, patterns of parental emotion expression may be altered in contexts of economic adversity, particularly when sociodemographic risk factors, such as low parental education, a history of being unhoused, or unemployment co-occur or accumulate (Evans et al., 2013). Cumulative social and economic adversity can increase the risk for emotional distress among parents and impair effective parenting behaviors (Carreras et al., 2019; Shaffer et al., 2012). Indeed, cumulative demographic risk has been associated with lower supportive parenting, as well as lower levels of positive expressiveness and greater negative expressiveness in families of young children, both using observed (Brophy-Herb et al., 2013) and self-reported (Raver & Spagnola, 2002) measures. Relatedly, parents experiencing poverty are more likely to be depressed compared to middle-class parents (Smith & Mazure, 2021), and depression has been linked to more negative and less positive emotional expression (Bylsma, 2020).

Risk processes for parent emotional expression may be particularly salient among homeless families, who tend to experience higher levels of poverty-related stress, cumulative demographic risks, and higher rates of parent psychopathology (Samuels et al., 2010). However, very few studies have directly assessed parental emotional expression among families experiencing homelessness. In one exception, Labella et al., (2023) found latent profiles of parental affect predicted child outcomes, such that parents showing elevated anger during observed-parent child interactions had children with greater observed difficulty downregulating anger and more teacher-reported social-behavioral problems at school. Given that healthy emotion expression among parents may play an important role in predicting adaptive development of low-income and housing-insecure children, further research is needed to clarify the links between poverty-related risk and emotion expression among parents experiencing homelessness. Furthermore, multi-method studies incorporating both self-reported and observer-rated measures of parental emotion expression are rare. Additional research is needed to clarify convergence between the two, especially in the context of acute adversity, which may contribute to heightened discrepancies between habitual behavior and current functioning.

## Parental Emotion Regulation Strategies in the Context of Poverty-Related Risk

Emotional experiences and expressions may be modulated by a wide range of regulatory processes, which have been commonly assessed through self-report measures of habitual strategy use (Aldao et al., 2010). In the context of family life, high quality parenting sometimes requires parents to downregulates the expression of negative emotions in order to de-escalate conflict and engage calmly with stressful parenting situations (Zalewski et al., 2018). This process often involves cognitive strategies that modulate thoughts to manage emotional reactivity, such as acceptance, positive refocusing, or positive reappraisal (Garnefski, et al., 2001; McRae, 2016). Generally, adaptive regulatory strategies are associated with higher life satisfaction and lower levels of depressed mood, as well as enhanced positive affect and decreased negative affect (Hu et al., 2014). Among parents, these adaptive strategies can function as a model for their children, which may help them develop their own regulatory skills (Crandall et al., 2015). Conversely, maladaptive regulatory strategies such as rumination, catastrophizing, or suppression, may be related to various negative outcomes, including higher incidence of psychopathology (Aldao et al., 2010; Garnefski et al., 2001; Gratz et al., 2018); parental psychopathology and mental health in turn significantly predict children’s emotional adjustment in both community samples (e.g., Cummings et al., 2005) and in samples characterized by high sociodemographic risk (e.g., Gewirtz et al., 2009). Furthermore, ineffective emotion regulation may directly affect parenting quality. For example, Hughes and Gullone (2010) found that mothers who reported suppressing their own emotions tended to minimize or punish their children’s negative emotions, whereas those who used positive reappraisal had children who showed lower levels of negative emotion expression.

Poverty-related stress may compound parenting challenges by prompting negative emotions and exhausting cognitive resources, making regulation more difficult (Zalewski et al., 2012). Additionally, parents in high-risk situations may put their emotional needs aside in favor of their children’s needs. For instance, in a mixed-methods study of families experiencing homelessness, mothers reported prioritizing parenting tasks over managing their own distress (Holtrop et al., 2015). Studies demonstrate that this sort of emotional suppression can be cognitively costly, leading to impaired memory and other negative physiological outcomes (Gross, 2015), while also paradoxically increasing the experience of negative emotions (Benita et al., 2019; Dalgleish et al., 2009). Conversely, maintaining a repertoire of adaptive regulatory strategies and minimizing reliance on maladaptive strategies may serve as a protective process for families in high-risk situations. For example, Palmer et al. (2020) found that, among families experiencing homelessness, lower parental use of maladaptive regulatory strategies in combination with high-quality parenting predicted lower internalizing symptoms in children. Given that parental regulatory strategies may serve a valuable protective function for developmental outcomes among children experiencing poverty and homelessness, it is important to further examine parental emotion regulation in the context of economic adversity.

## Current Study

The current study addressed gaps in the existing literature by examining the relationships between parental emotion experience, expression, and regulation in the context of homelessness and high sociodemographic risk. Prior studies from this research team have documented links between aspects of emotion socialization and child outcomes among families experiencing homelessness ( Labella et al., 2016; Palmer et al., 2020). The present study is the first to focus on intrapersonal associations among emotion-related variables in parents, and the first to analyze potential links among emotional experience, emotional expression, and regulatory strategy use. This approach is particularly important because previous research with families experiencing homelessness unexpectedly found that self-reported emotional experience and observed affect were largely unrelated, specifically during a parent-child interaction task (Palmer et al., 2022). The present study also extended prior work through a multi-method assessment of parental emotion expression, including self-reports and observer ratings. Clarifying associations among emotional experience, expression, and regulation in parents experiencing poverty and homelessness may help to inform interventions designed to enhance resilience across generations.

We hypothesized that (1) parental emotional experiences would predict emotion expression in valence-specific ways. Specifically, we expected that *experience* of positive and negative emotion would be linked with the *expression* of positive and negative emotion, respectively. Secondly, we expected that (2) parental emotion regulation would predict emotion expression while controlling for the effects of emotional experience and relevant sociodemographic covariates. We anticipated that more adaptive emotion regulation strategy use would be associated with more positive and less negative emotion (both experienced and expressed), whereas maladaptive emotion regulation strategies would be associated with more negative and less positive emotion. Finally, we hypothesized that (3) parental emotion regulation would moderate the links between the experience and expression of negative emotion. We expected that adaptive emotion regulation would weaken the link between the experience and expression of negative affect, whereas maladaptive emotion regulation would strengthen that link. Exploratory analyses were additionally planned to examine the potential moderation of negative affect by emotion regulation in predicting positive expression.

## Method

### Participants

Participants were recruited using fliers in mailboxes and informational tables during mealtimes at two emergency family shelters in a Midwestern city. Families with a child expected to enter kindergarten or first grade during the subsequent school year were eligible to participate after they had lived in shelter for at least three days to allow time for adjusting to the shelter. Of 238 families who met initial criteria, 180 families were eligible; the remainder were excluded due to insufficient English to complete study measures (*n* = 55) or severe developmental delays interfering with study completion (*n* = 3). Of the eligible families, 110 consented to participate, 8 declined, and 62 could not be scheduled, missed their scheduled session, or were not in contact with study staff. Two parents who consented had scheduling conflicts preventing completion of study activities, resulting in a final sample of 108 primary caregivers with 4- to 6-year-old children (58.2% male, *M*_*age*_ = 5.8 years, *SD* = 0.6) residing in emergency family housing.

Primary caregivers identified primarily as female (89.8%) with an average age of 30.3 years old (SD = 6.6). Most were biological mothers (88.0%) of the target child, and the remainder were biological fathers (8.3%), grandmothers (1.9%), or stepparents (1.9%). Most parents were Black/African/African American (61.2%), with the remainder identifying as American Indian/Alaska Native (14.0%), Multiracial (11.2%), White (9.3%), or Asian/Pacific Islander (3.7%). The median number of nights spent in emergency housing prior to assessment was 17 and the mean was 44.6 (*SD* = 78.9); although most families reported having been in emergency housing between three nights and three months, the distribution was positively skewed by three families who had remained in shelter for longer than a year.

### Procedure

The University of Minnesota IRB approved all study procedures. Study sessions took place in private research rooms on nonresidential floors inside the emergency shelters. Parents provided informed consent and children provided verbal assent. Children participated in an assessment of school readiness skills while parents were interviewed about demographics, child behavior, and stressful life events. Following individual sessions, parents and children participated in a structured sequence of Family Interaction Tasks (DeGarmo et al., 2004), which were previously adapted and abridged for use with homeless families (Gewirtz et al., 2009; Narayan et al., 2012). Interaction tasks were video recorded for later coding of parent emotion expression. Parents received an honorarium and children received a small toy for their time.

### Measures

#### Parent Emotional Experiences

Parental emotional experience was measured with the Positive and Negative Affect Schedule, a well-validated and widely used measure of subjective emotion (PANAS; Watson et al., 1988). Items consist of a single word describing a discrete positive (e.g., enthusiastic, proud) or negative (e.g., upset, hostile) emotion. For each emotion, respondents were instructed to “indicate to what extent you have felt this way during the past few weeks” on a 5-point scale (1 = *very slightly or not at all*, 5 = *very much*). Items were summed to form two 10-item scales capturing positive (α = 0.87) and negative emotional experiences (α = 0.83), respectively.

#### Self-reported Emotion Expression

Self-reported emotion expression was measured using the Self-Expressiveness in the Family Questionnaire: Short Form (SEFQ-SF), a reliable and valid questionnaire measure of habitual emotional expressiveness in the family context (Halberstadt et al., 1995). Participants indicated how often they expressed specific positive (e.g., “expressing thanks to someone for doing you a favor”) or negative emotions (e.g., “breaking down emotionally when you’re under a lot of stress”) with family members on a 9-point scale (1 = *Not at all frequently*, 9 = *Very frequently*). Items were summed to form overall scores for positive (α = 0.81) and negative emotional expressiveness (α = 0.76).

#### Observed Emotion Expression

Parents’ observed emotion expression was rated from two video-recorded interaction tasks, a problem-solving discussion and a marble maze game. These tasks were selected because they were expected to elicit a range of emotion expressions: predominantly negative affect during the problem-solving discussion and predominantly positive affect during the collaborative game. Parent affect was coded using a microsocial coding system adapted from manuals used in prior research on parent-child interaction (Morris et al., 2011; Cui et al., 2015). Each task took five to six minutes, resulting in 10 to 12 min of interaction data. Independent coding teams rated intensity of a single affect (anger, internalizing distress, or positive affect) on a five-point scale from *1–none* to 5–*very strong* in ten-second intervals across each task. Indicators of anger included crossed arms, raised voices, and lowered brows; indicators of distress included anxious fidgeting, downcast eyes, and tearful voices; and indicators of positive affect included smiling, laughter, and physical celebrations (e.g., high fives). The senior author served as anchor coder for all affect coding teams. Ten undergraduate, graduate, and postbaccalaureate coders achieved good reliability with the anchor coder [*Intraclass correlation* (*ICC)* ≥ 0.75] on practice recordings before coding for the present study. Twenty percent of video recordings were randomly selected for double coding. Discrepancies were discussed and resolved by consensus at weekly reliability meetings to minimize rater drift. When available, consensus codes were used for analyses. Inter-rater reliability ranged from moderate to good (anger and distress: *ICC* = 0.70, positive: *ICC* = 0.86). Individual intervals were coded as missing if there was no evidence of a given affect *and* the parent was not codable at least half the interval (e.g., moved off screen). Mean affect intensities were calculated for a given task if at least 20 intervals were codable, to ensure that composites were drawn from relative stable estimates, resulting in a sample size of *n* = 102 for both negative affect codes during the discussion and positive affect codes during the game.

Mean affect intensities were calculated for each task and compared using paired-samples t-tests. As expected, parents expressed more distress and anger during the problem-solving discussion than the game (distress: discussion *M* = 1.80, *SD* = 0.35, vs. game *M* = 1.49, *SD* = 0.27, *t*[94] = 8.98, *p* < 0.001; anger: discussion *M* = 1.35, *SD* = 0.23 vs. game *M* = 1.23, *SD* = 0.18, *t*[86] = 4.99, *p* < 0.001). Conversely, parents expressed more positive affect during the game than the discussion (discussion *M* = 1.83, *SD* = 0.52 vs. game *M* = 2.26, *SD* = 0.50, *t*[95] = −7.66, *p* < 0.001). Analyses focused on observed positive and negative affect during interaction tasks conducive to positive and negative emotions, respectively. Thus, we focused on parental positive affect during the game and parental negative affect during the discussion. Mean affect intensities of parental anger and distress during the problem-solving discussion were averaged to form a single measure of parental negative emotion expression to parallel the Self-Expressiveness in the Family Questionnaire, which includes a single factor for both negative dominant (e.g., anger) and negative submissive (e.g., distress) emotions.

#### Parent Emotion Regulation Strategy use

Emotion regulation (ER) was measured with two variables: adaptive ER and maladaptive ER. Parents reported on regulatory strategies using an abbreviated version of the Cognitive Emotion Regulation Questionnaire (CERQ; Garnefski et al., 2001), a well-established and empirically tested measure of habitual ER strategies. Habitual ER has itself been linked to momentary emotion reactivity (Maxwell et al., 2018). The CERQ is composed of nine subscales of four items each. To reduce participant burden, five subscales (positive reappraisal, positive refocusing, self-blame, rumination, and catastrophizing) were selected for administration in the current study based on theory and research supporting their relevance for psychological wellbeing (Aldao et al., 2010; Aldao & Nolen-Hoeksema, 2010). When the CERQ was psychometrically validated, the selected subscales showed significant unique associations with depression and anxiety, controlling for all other strategies, whereas the other four subscales did not (Garnefski et al., 2001). Based on the direction of these associations, supported by related theory and research (Aldao et al., 2010; Aldao & Nolen-Hoeksema, 2010), positive reappraisal and positive refocusing were identified as adaptive strategies, and self-blame, rumination, and catastrophizing were identified as maladaptive strategies.

Parents rated their likelihood of engaging in specific regulatory strategies in response to stressful life events on a 5-point scale from 1-*Almost Never* to 5-*Almost Always*. Adaptive strategies were measured by subscales tapping positive reappraisal (a = 0.72, e.g., “you think that situation also has its positive sides”) and positive refocusing (a = 0.72, e.g., “you think of nicer things than what you have experienced”). Maladaptive strategies were measured by subscales assessing self-blame (a = 0.79, e.g., “you feel that you are the one to blame for it”), rumination, (a = 0.75, e.g., “you dwell upon the feelings the situation has brought up for you”), and catastrophizing (a = 0.75, e.g., “you continually think how horrible the situation has been”). Items were summed within subscales and averaged to form composites indexing adaptive (a = 0.80) and maladaptive (a = 0.85) emotion regulation strategies.

#### Sociodemographic Risk

Parents reported sociodemographic information, including their level of education, employment status, and history of homelessness. Parental reports were used to derive a cumulative sociodemographic risk score, calculated as the sum of ten binary risk factors previously identified as relevant to families experiencing severe and poverty and homelessness (Evans et al., 2013; Obradović et al, 2012; Labella et al., 2019). Risk factors were as follows: single parent; four or more children in the household; parent under 18 at birth of first child; primary caregiver has less than high school education; primary caregiver unemployed; family unable to afford rent; lived in substandard housing; lived in unsafe neighborhood; target child has lived at five or more addresses; and primary caregiver homeless three or more times.

#### Demographic Covariates

Parents reported their own age and sex, as well as the age and sex of their child.

### Plan for Analysis

Analyses were conducted in IBM SPSS Statistics Version 27. We evaluated bivariate correlations of emotion-related variables with demographic covariates and sociodemographic risk, with the intention of including significant predictors in our focal analyses. Focal analyses were a series of three-step hierarchical linear regressions for each relevant emotion expressiveness outcome variable (self-reported negative; self-reported positive; observed negative; observed positive). Because emotional experience theoretically involves an internal feeling whose outward expression is modulated by emotion regulation, positive and negative emotional experience were entered on the first step, along with candidate covariates that were significant at the bivariate level. Adaptive and maladaptive emotion regulation strategies were entered on the second step, and interactions between negative emotional experiences and each regulatory strategy variable were entered in separate models (steps 3a and 3b).

#### Missing Data

The proportion of missing data was minimal, ranging from 0% (sociodemographic variables) to 5.6% (parent affect). Little’s test was used to test the assumption data were missing completely at random (MCAR; Little, 1988). Results were not statistically significant (χ²[45, *N* = 108] = 48.04, *p* = 0.35), permitting the assumption that data were MCAR. We used multiple imputation with the package ‘mice’ (van Buuren & Groothuis-Oudshoorn, 2011) in R version 4.1.3 (R Core Team, 2021) to generate unbiased parameter estimates across 25 datasets using fully conditional specifications, which were pooled according to Rubin’s rules (Rubin, 2009). Approximate effect sizes and significance judgments were comparable using raw and imputed data. Given the small amount of missing data, results using raw data are reported.

## Results

### Preliminary Analyses

Descriptive statistics and bivariate correlations are presented in Table 1. Parents tended to report higher levels of positive emotion experience and expression compared to negative emotions. Positive and negative observed affect showed means of 2.25 and 1.80, respectively on a 1 to 5 scale. Ratings of 2 indicate *Low or ambiguous* levels of a given affect in the coded interval. Thus, task averages for positive and negative emotions expressions may indicate consistent low levels (e.g., frequent restrained expressions of negative emotion during the conflict discussion) and/or patterns of fluctuation (e.g., clear expressions of joy alternating with neutral affect while concentrating on the game).

**Table 1:** Descriptive statistics and bivariate correlations for focal variables and demographics

	1	2	3	4	5	6	7	8	9	10	11
1. Sociodemographic risk	--										
2. Parent age	−0.12	--									
3. Child age	−0.20*	0.13	--								
4. Negative emotional experience	0.13	0.06	−0.25**	--							
5. Positive emotional experience	−0.12	−0.08	−0.20*	−0.26**	--						
6. Self-reported negative expressiveness	0.14	−0.03	0.06	0.30**	−0.19	--					
7. Self-reported positive expressiveness	−0.10	0.14	0.07	0.02	0.31**	−0.02	--				
8. Maladaptive regulatory strategy use	0.05	−0.04	−0.04	0.49***	−0.14	0.28**	0.11	--			
9. Adaptive regulatory strategy use	−0.09	−0.05	0.12	−0.13	0.58**	−0.25**	0.44***	0.06	--		
10. Observed negative expressiveness	−0.12	0.02	0.11	0.06	0.00	0.05	0.08	0.14	−0.05	--	
11. Observed positive expressiveness	0.03	−0.15	0.08	−0.16	0.12	0.01	0.22*	−0.00	0.30**	−0.04	--
Mean	3.99	30.31	5.70	24.63	37.09	46.73	83.15	32.95	29.63	1.80	2.25
Standard deviation	5.70	6.56	0.60	7.53	7.55	14.06	14.93	9.30	5.98	0.35	0.49
Minimum-Maximum	1–9	19–50	4.50–6.92	11–46	14–50	19–83	12–108	15–58	16–40	1.12–2.43	1.00–3.40

* p < 0.05, ** p < 0.01, *** p < 0.001

Preliminary analyses examined whether demographic characteristics of participants and their children were significantly correlated to parental emotion. Older child age was weakly associated with more positive (*r* = 0.20, *p* = 0.042) and less negative (*r* = −0.25, *p* = 0.009) emotional experience. Female caregivers showed more negative emotion (*M* = 1.66, *SD* = 0.27) than male caregivers (*M* = 1.54, *SD* = 0.10, *t*[32.61] = 3.05, *p* = 0.005); results should be interpreted with caution given the small number of male caregivers in the sample (*N* = 11). Sociodemographic risk was not significantly related to any parental emotion variable and was excluded from further analyses.

As expected, parents’ self-reported emotional experiences were associated with self-reported expressiveness in valence-specific ways (positive *r* = 0.31, *p* = 0.001; negative *r* = 0.30, *p* = 0.002). Only self-reported positive expressiveness predicted observed positive expressiveness during a collaborative game (*r* = 0.22, *p* = 0.024); negative self-reported expressiveness was not related to observed expressiveness during structured parent-child interactions. Regarding bivariate associations with emotion regulatory strategies, adaptive ER was positively associated with positive emotional experiences (*r* = 0.58, *p* < 0.001), self-reported positive expressiveness (*r* = 0.44, *p* < 0.001), and observed positive expressiveness during the game (*r* = 0.30, *p* = 0.002). Adaptive ER was negatively correlated with self-reported negative expressiveness (*r* = −0.25, *p* < 0.001), but was not significantly associated with self-reported negative emotional experiences or observed negative expressiveness during a problem-solving discussion. Maladaptive ER was positively associated with negative emotional experiences (*r* = 0.53, *p* < 0.001) and self-reported negative expressiveness (*r* = 0.25, *p* = 0.012), but not observed negative expressiveness during a problem-solving discussion. Maladaptive ER was not related to positive emotion experience or expression.

### Focal Analyses

Hierarchical linear regression models tested for the main and interaction effects of emotional experiences and ER strategies as predictors of emotional expression. Based on bivariate associations, child age was included as a covariate in all models and parent sex was included as a covariate in models predicting observed negative expressiveness. When predicting self-reported negative expressiveness (Table 2), we found that negative emotional experiences, but not positive emotional experiences or child age, significantly predicted self-reported negative expressiveness with a small-to-moderate effect (β = 0.31, *p* = 0.002). The addition of ER terms in Step 2 led to a significant increment in R^2^ (*F*[2, 98] = 3.64, *p* = 0.03), accounting for an additional 6.0% of variance in self-reported negative expressiveness. Adaptive (but not maladaptive) ER significantly predicted negative expressiveness with a small-to-moderate effect (β = −0.26, *p* = 0.025), and negative emotional experience was no longer a significant predictor. The addition of interaction terms between negative emotional experiences and adaptive ER (on Step 3 A) or maladaptive ER (on Step 3B) did not account for significant increases in variance explained.

**Table 2:** Hierarchical linear regression predicting parents' self-reported negative emotional expressiveness from emotional experience and regulation

	*B*	*SE B*	*β*	R^2^	F for ΔR^2^
*Parental self-reported negative emotional expressiveness*					
1. (Intercept)	−1.39	0.94		0.13	4.90**
Negative emotional experience	0.31**	0.10**	0.31**		
Positive emotional experience	−0.14	0.10	−0.14		
Child age	0.25	0.16	0.15		
2. (Intercept)	−1.23	0.92		0.19	3.64*
Negative emotional experience	0.21	0.11	0.21		
Positive emotional experience	0.02	0.12	0.02		
Child age	0.22	0.16	0.13		
Maladaptive regulation	0.20	0.11	0.20		
Adaptive regulation	−0.26*	0.11*	−0.26*		
3a. (Intercept)	−1.29	0.92		0.19	0.43
Negative emotional experience	0.21	0.11	0.21		
Positive emotional experience	0.03	0.12	0.03		
Child age	0.23	0.16	0.14		
Maladaptive regulation	0.18	0.11	0.18		
Adaptive regulation	−0.25*	0.12*	−0.25*		
Negative affect × Adaptive regulation	−0.06	0.08	−0.07		
3b. (Intercept)	−1.24	0.91		0.21	2.30
Negative emotional experience	0.30	0.12	0.30		
Positive emotional experience	0.06	0.12	0.06		
Child age	0.23	0.16	0.14		
Maladaptive regulation	0.19	0.11	0.19		
Adaptive regulation	−0.31**	0.12**	−0.32**		
Negative affect × Maladaptive regulation	−0.14	0.09	−0.17		

* p < 0.05, ** p < 0.01

Similar patterns were observed when self-reported positive expressiveness (Table 3). Positive emotional experiences, but not negative emotional experiences or child age, significantly predicted self-reported positive expressiveness with a small-to-moderate effect (β = 0.33, *p* = 0.001). After adding ER terms in Step 2, adaptive ER emerged as a significant predictor with a moderate effect (β = 0.38, *p* = 0.001). The addition of ER accounted for an additional 10.6% of variance in positive expressiveness, a significant increase in explained variance (*F*[2, 98] = 6.59, *p* = 0.002). Neither adaptive ER (Step 3A) nor maladaptive ER (Step B) moderated the relation of negative emotional experiences with self-reported positive expressiveness.

**Table 3:** Hierarchical linear regression predicting parents' self-reported positive emotional expressiveness from emotional experience and regulation

	*B*	*SE B*	*β*	R^2^	F for ΔR^2^
*Parental self-reported positive emotional expressiveness*					
1. (Intercept)	−0.30	0.96		0.11	3.99*
Negative emotional experience	0.12	0.10	0.12		
Positive emotional experience	0.33**	0. 10**	0.33**		
Child age	0.05	0.17	0.03		
2. (Intercept)	−0.20	0.91		0.21	6.59**
Negative emotional experience	0.08	0.11	0.08		
Positive emotional experience	0.11	0.12	0.11		
Child age	0.04	0.16	0.02		
Maladaptive regulation	0.07	0.12	0.07		
Adaptive regulation	0.38**	0.11**	0.38**		
3a. (Intercept)	−0.32	0.91		0.23	1.76
Negative emotional experience	0.08	0.11	0.08		
Positive emotional experience	0.15	0.12	0.15		
Child age	0.05	0.16	0.03		
Maladaptive regulation	0.04	0.11	0.04		
Adaptive regulation	0.40**	0.11**	0.40**		
Negative affect × Adaptive regulation	−0.11	0.08	0.13		
3b. (Intercept)	−0.20	0.98		0.22	0.22
Negative emotional experience	0.05	0.13	0.05		
Positive emotional experience	0.10	0.12	0.10		
Child age	0.03	0.16	0.02		
Maladaptive regulation	0.07	0.12	0.07		
Adaptive regulation	0.40**	0.12**	0.40**		
Negative affect × Maladaptive regulation	0.04	0.09	0.05		

* p < 0.05, ** p < 0.01

The model predicting observed negative expressiveness was not statistically significant at any step (Table 4). Consistent with bivariate findings, observed negative expressiveness was not significantly associated with emotional experiences or child age on Step 1, nor with ER strategies on Step 2. There was no evidence of a link between emotional experience and observed negative expressiveness moderated by adaptive or maladaptive ER.

**Table 4:** Hierarchical linear regression predicting parents' observed negative emotional expressiveness from emotional experience and regulation

	*B*	*SE B*	*β*	R^2^	F for ΔR^2^
*Observed negative parental emotional expressiveness*					
1. (Intercept)	−1.42	1.03		0.05	1.16
Negative emotional experience	0.08	0.11	0.08		
Positive emotional experience	0.00	0.11	0.00		
Child age	0.26	0.18	0.15		
Parent sex	−0.56	0.34	0.15		
2. (Intercept)	−1.26	1.04		0.06	0.89
Negative emotional experience	0.02	0.12	0.02		
Positive emotional experience	0.07	0.13	0.07		
Child age	0.232	0.18	0.13		
Parent sex	−0.55	0.34	−0.16		
Maladaptive regulation	0.14	0.12	0.14		
Adaptive regulation	−0.11	0.13	−0.11		
3a. (Intercept)	−1.32	1.04		0.07	0.99
Negative emotional experience	0.02	0.12	0.02		
Positive emotional experience	0.10	0.13	0.10		
Child age	0.24	0.18	0.14		
Parent sex	−0.52	0.34	−0.16		
Maladaptive regulation	0.12	0.12	0.11		
Adaptive regulation	−0.10	0.13	−0.10		
Negative affect × Adaptive regulation	−0.09	0.09	−0.11		
3b. (Intercept)	−1.20	1.04		0.08	1.25
Negative emotional experience	−0.05	0.14	−0.05		
Positive emotional experience	0.04	0.13	0.04		
Child age	0.21	0.18	0.12		
Parent sex	−0.52	0.34	−0.16		
Maladaptive regulation	0.15	0.12	0.15		
Adaptive regulation	−0.06	0.13	−0.06		
Negative affect × Maladaptive regulation	0.12	0.10	0.13		

The model predicting observed positive expressiveness is presented in Table 5. Consistent with bivariate findings, observed positive expressiveness was not significantly associated with positive emotional experiences, negative emotional experiences, or child age on Step 1. The addition of ER on Step 2 resulted in a statistically significant change in R^2^ (*F*[2, 94] = 4.60, *p* = 0.012), accounting for an additional 8.2% of variance; variance was accounted for principally by adaptive ER (β = 0.35, *p* = 0.004; a moderate effect). The relationship between negative emotional experiences and self-reported positive expressiveness was not moderated by adaptive ER (Step 3 A) or maladaptive ER (Step 3B).

**Table 5:** Hierarchical linear regression predicting parents' observed positive emotional expressiveness from emotional experience and regulation

	*B*	*SE B*	*β*	R^2^	F for ΔR^2^
*Observed positive parental emotional expressiveness*					
1. (Intercept)	−0.19	0.98		0.04	1.16
Negative emotional experience	−0.14	0.11	−0.14		
Positive emotional experience	0.09	0.10	0.09		
Child age	0.04	0.17	0.02		
2. (Intercept)	−0.22	0.96		0.12	4.60*
Negative emotional experience	−0.17	0.12	−0.17		
Positive emotional experience	−0.11	0.121	−0.11		
Child age	0.04	0.17	0.02		
Maladaptive regulation	0.02	0.11	0.02		
Adaptive regulation	0.34*	0.12*	0.02*		
3a. (Intercept)	−0.23	−0.97		0.12	0.04
Negative emotional experience	−0.17	0.12	−0.17		
Positive emotional experience	−0.11	0.13	−0.11		
Child age	0.04	0.17	0.02		
Maladaptive regulation	0.02	0.11	0.02		
Adaptive regulation	0.35*	0.12*	0.02*		
Negative affect × Adaptive regulation	−0.02	0.09	−0.02		
3b. (Intercept)	0.25	0.96		0.13	0.76
Negative emotional experience	−0.12	0.13	−0.12		
Positive emotional experience	−0.09	−0.12	−0.09		
Child age	0.05	0.17	0.03		
Maladaptive regulation	0.02	0.11	0.02		
Adaptive regulation	0.31*	0.12*	0.32*		
Negative affect × Maladaptive regulation	−0.08	0.10	−0.10		

* p < 0.05, ** p < 0.01

## Discussion

The present study aimed to examine associations among emotional experience, expression, and regulation among parents of young children experiencing homelessness. We tested bivariate relationships between emotional experience and expression, both self-reported and observed. Consistent with our expectations, we found that self-reported positive and negative emotional experiences were related to self-reported expressiveness in valence-specific ways. Contrary to our expectations, emotional experiences were not related to observed emotion expressions. In other words, internal experiences of emotion were related to self-perceptions of expression but did not predict independently observed outward expression. Our measures of self-reported and observed expressiveness, while conceptually related, may differ given that the SEFQ assesses habitual emotion expressiveness, whereas observed emotion expression measured affect during a single set of interactions elicited during a research protocol. This is counter to previous research in which habitual measures do tend to be related to momentary observed measures (e.g., Maxwell et al., 2018; Wylie et al., 2022). Interestingly, higher self-reported positive expressiveness predicted higher observed positive expressiveness, as expected, whereas self-reported and observed measures of negative expressiveness were not related. This disconnect may reflect successful downregulation of negative emotion during stressful parent-child interactions, consistent with our expectations that more adaptive emotion regulation would be related to lower expression of negative emotion. Given that positive expressiveness is generally considered more socially acceptable than negative expressiveness, parents in our sample may have tended to under-report their habitual expression of negative emotion and/or minimize outward expression of distress and frustration during video-recorded interactions. It is also possible that the motivation to minimize the expression of negative emotions may also have affected the general null findings associated with observed negative emotion – the only variable which was not significantly associated with any independent variables.

At the bivariate level, we found that adaptive regulatory strategies were related to more positive and less negative emotional experience, as well as more positive observed and self-reported emotional expressiveness. This result is consistent with previous findings linking adaptive regulatory strategies to increased positive affect and decreased negative affect (e.g., Hu et al., 2014). Adaptive emotion regulation therefore seems to be broadly related to greater expression of positive emotions and may support the downregulation of negative emotional experience. Bivariate correlations involving maladaptive emotion regulation revealed positive associations with both negative emotional experience and self-reported negative expressiveness, consistent with previous findings that maladaptive strategies are related to more negative emotion (e.g., Wante et al., 2018).

We hypothesized that emotion regulation would predict both self-reported and observed emotion expression over and above the effects of emotional experience and relevant sociodemographic covariates, and that emotion regulation would moderate the links between the experience and expression of negative emotion. Our first hypothesis was partially supported. Adaptive ER, but not maladaptive ER, significantly predicted both positive and negative self-reported emotion expressiveness and observed positive emotion expression, above and beyond the variance accounted for by emotional experience. Notably, emotional experience no longer predicted self-reported emotion expressiveness when controlling for regulatory processes, implying shared variance accounted for by adaptive ER. Although causal direction cannot be determined, this suggests that effective regulation may facilitate more positive and less negative outward emotional expressions in part by contributing to more positive and less negative internal experience. In contrast, our analyses did not demonstrate moderation of the link between negative emotional experience and emotional expressiveness by emotion regulatory strategy use. Taken together, results suggest that the ability to maintain adaptive self-regulatory strategies, such as positive reappraisal and positive refocusing of attention, may help parents to maintain and express positive emotions, even amid substantial psychosocial stress. Adaptive strategies appeared to function as a promotive factor (main effect), regardless of the level of negative emotional experiences that parents reported, rather than moderating the effect of negative emotions.

Consistent with bivariate findings, our regression model for observed negative expressiveness identified no unique predictors. There may have been more proximate predictors to the parents’ negative affect that were not included in our study (e.g., current mood, general levels of stress, and child’s behavior), but this result may also reflect a tendency for participants to downplay or minimize negative expression during recorded interactions due to the stigma related to negative emotions. Indeed, there appears to be generally low coherence between negative emotion experience and observed negative emotion expression across the literature (Reisenzein et al., 2013), such that our null findings are not unique to our sample. Future research is needed to clarify predictors (e.g., adverse life experiences, parental psychopathology) of observed negative expressiveness among parents who may be vulnerable to negative emotionality due to severe poverty and homelessness.

Notably, of the demographic covariates we tested, only child age and parent sex had significant associations with emotional experience and observed emotional expression, respectively. Older child age was associated with more positive and less negative emotional experience, whereas female caregiver sex was associated with greater observed negative expressiveness. The latter finding should be interpreted with caution pending replication in samples with more even distributions of male and female caregivers. Degree of sociodemographic risk was not significantly related to parents’ positive or negative emotion expression. This may reflect the generally high-risk nature of this population; indeed, in a similar sample, sociodemographic risk was not significantly related to parents’ warmth or negativity when discussing their children (Labella et al., 2016).

### Strengths and Limitations

To our knowledge, this study is the first to analyze the links between intrapersonal emotional experience and emotion regulatory strategies with parental emotion expression in a population of families experiencing homelessness. Extreme low-income groups are often overlooked by scientific research (Polo et al., 2019). However, people in these environments are particularly susceptible to negative mental health and emotion outcomes (Evans et al., 2013; Wiewel & Hernandez, 2021). Given the overrepresentation of racial and ethnic minorities in populations of economic adversity (U.S. Department of Housing and Urban Development, 2024) and the health disparities associated with race and ethnicity (Dressler et al., 2005), the present study helps to fill a key gap in the literature. In particular, results could inform strategies to foster resilience in caregivers and children experiencing homelessness, specifically by encouraging the use of adaptive regulation strategies, and working to increasing habitual experiences of positive emotions. An additional strength of this study is the multi-method assessment of emotion expressiveness. Given the unexpected findings of non-significant relation between negative self-reported and observed expression, our study highlights the need to consider the method of data acquisition in how we interpret key findings of emotion expression. One additional consideration is the nature of our measure of emotion regulation, which assesses *habitual* use of adaptive and maladaptive strategies. Critically, past research has demonstrated that habitual strategy use is associated with momentary self-reported emotion reactivity and experience (Maxwell et al., 2018). Nonetheless, there is a notable lack of research establishing such a connection with observed behaviors, especially among marginalized groups. Our study is among the few to examine the connection between habitual emotion regulation measures and multi-method measures of momentary emotionality in a sample characterized by high sociodemographic risk.

This study is limited in its relatively small sample size (*N* = 108), which is underpowered to detect small interaction effects. Thus, null findings related to moderation await further study in larger samples. For the purposes of this study, we relied on parental self-report for emotional experiences and habitual regulatory use, which may amplify statistical associations due shared method variance. This study would have benefitted from additional measures of these constructs, including physiological measures associated with emotional experience and regulation. Finally, this study employed a cross-sectional and correlational design, which limits the ability to draw inferences about the direction of the relationship between emotional experiences, expression, and regulatory strategies.

### Future Directions and Conclusions

Our findings demonstrated that *adaptive* regulatory strategies predict parental emotion expression above and beyond the *experience* of emotion. Additionally, we confirmed that adaptive regulatory strategies are associated with more positive emotional experience and less negative emotional experience. These results are broadly consistent with therapeutic frameworks that aim to enhance emotional wellbeing by targeting responses to emotional experiences (Gratz et al., 2015). However, we did not find moderating effects of emotion regulation on the link between experience and expression of emotion, as we had hypothesized. Further examination of these intrapersonal emotional relationships is needed in contexts of high sociodemographic risk. Other potentially relevant covariates not assessed by this study, including parent or child psychopathology and child temperament, may influence the capacity for parents to effectively incorporate regulatory strategies in various parenting contexts (Kiff et al., 2011; Zimmer-Gembeck & Skinner, 2016). These individual differences may predict parents’ emotional functioning. Additional work also is needed to examine similar interrelations among emotion-related attributes among children as well as their parents.

Another important direction for future research is interventions to foster resilience among parents and children through emotion regulation strategies. While many interventions focus on children in school environments (e.g., Durlak et al 2011), children’s emotion socialization is largely shaped by their parents’ modeling of emotions and emotion regulation (Crandall et al., 2015; Zinsser et al 2021). Therefore, interventions that seek to improve regulatory strategies and emotion modeling among high-risk parents may be able to improve those parents’ current wellbeing (Aldao et al., 2010; Balzarotti et al., 2016; Garnefski et al., 2001) while also improving emotion socialization behaviors. This could have significant downstream effects on their children’s wellbeing, helping to interrupt intergenerational patterns of poor mental health outcomes. Based on our findings, such interventions might target supportive adaptive ER among high-risk parents, given its predictive capacity over the experience of more positive (and less negative) emotion, which itself was related to more positive emotion expression. Furthermore, qualitative research may be helpful to identify individual adaptive ER strategies that work well for high-risk parents and families.

It is important to note that the present study included predominantly Black families experiencing homelessness, and there is evidence that emotion socialization processes may be altered by a history of poverty and racism. An integrative framework of socialization in ethnic minority families postulates that racial/ethnic socialization and emotion socialization occur simultaneously and overlap among such families (Dunbar et al., 2017). Black parents may, for example, respond restrictively to emotional displays in an effort to prepare their children for future experiences of discrimination (Dunbar et al., 2017). While we believe it to be a strength to include underrepresented groups in research, inconsistent results in similar research may be due to differences in cultural contexts. Further research is necessary to clarify the role of racial and ethnic contexts in emotion socialization across different cultural groups, and such research should approach this with a culturally responsive lens that understands how ethnic/racial socialization interacts with emotion socialization.

### Summary

Children who experience economic adversity are especially vulnerable to a range of negative mental health, academic, and economic outcomes (Bassuk, 2010; Evans et al., 2013). Given that adaptive emotion socialization may serve as a protective factor for children in high-risk environments, it is particularly important to understand the factors associated with parental modeling of emotion. The current study represents an important step toward clarifying the processes connecting emotion experience, expression, and regulation among parents experiencing homelessness. Future research on emotion expression and regulation could guide the development of interventions that help parents incorporate adaptive emotion regulation strategies to enhance resilience across generations in families experiencing poverty and residential instability.
